# Emerging spatial patterns in Antarctic prokaryotes

**DOI:** 10.3389/fmicb.2015.01058

**Published:** 2015-09-30

**Authors:** Chun-Wie Chong, David A. Pearce, Peter Convey

**Affiliations:** ^1^Department of Life Sciences, School of Pharmacy, International Medical University, Kuala LumpurMalaysia; ^2^National Antarctic Research Center, University of Malaya, Kuala LumpurMalaysia; ^3^Faculty of Health and Life Sciences, University of Northumbria, Newcastle upon TyneUK; ^4^University Centre in Svalbard, LongyearbyenNorway; ^5^British Antarctic Survey, CambridgeUK

**Keywords:** spatial pattern, Antarctica, prokaryotes, functional redundancy, biogeography

## Abstract

Recent advances in knowledge of patterns of biogeography in terrestrial eukaryotic organisms have led to a fundamental paradigm shift in understanding of the controls and history of life on land in Antarctica, and its interactions over the long term with the glaciological and geological processes that have shaped the continent. However, while it has long been recognized that the terrestrial ecosystems of Antarctica are dominated by microbes and their processes, knowledge of microbial diversity and distributions has lagged far behind that of the macroscopic eukaryote organisms. Increasing human contact with and activity in the continent is leading to risks of biological contamination and change in a region whose isolation has protected it for millions of years at least; these risks may be particularly acute for microbial communities which have, as yet, received scant recognition and attention. Even a matter apparently as straightforward as Protected Area designation in Antarctica requires robust biodiversity data which, in most parts of the continent, remain almost completely unavailable. A range of important contributing factors mean that it is now timely to reconsider the state of knowledge of Antarctic terrestrial prokaryotes. Rapid advances in molecular biological approaches are increasingly demonstrating that bacterial diversity in Antarctica may be far greater than previously thought, and that there is overlap in the environmental controls affecting both Antarctic prokaryotic and eukaryotic communities. Bacterial dispersal mechanisms and colonization patterns remain largely unaddressed, although evidence for regional evolutionary differentiation is rapidly accruing and, with this, there is increasing appreciation of patterns in regional bacterial biogeography in this large part of the globe. In this review, we set out to describe the state of knowledge of Antarctic prokaryote diversity patterns, drawing analogy with those of eukaryote groups where appropriate. Based on our synthesis, it is clear that spatial patterns of Antarctic prokaryotes can be unique at local scales, while the limited evidence available to date supports the group exhibiting overall regional biogeographical patterns similar to the eukaryotes. We further consider the applicability of the concept of “functional redundancy” for the Antarctic microbial community and highlight the requirements for proper consideration of their important and distinctive roles in Antarctic terrestrial ecosystems.

## Introduction

Due to their importance to the fundamental assembly of ecosystems, considerable effort has been devoted to study of the interactions of spatial scale, external physicochemical parameters and species distributions (e.g., King et al., 2010; Nemergut et al., 2011; Westgate et al., 2014). Spatial patterns of species distribution arise from the interactions between physical, chemical, and biological drivers (Legendre and Fortin, 1989; Prosser et al., 2007), placed in the context of the past regional colonization and evolutionary history of any given region (Convey et al., 2014). From the physical environment perspective, environmental gradients clearly influence the establishment and maintenance of viable populations; however, the spatial scale considered is also important in defining these environmental gradients (Wiens, 1989). For instance, in soils, environmental parameters at micro-scale are strongly correlated with the soil texture, pore space, and local topography (e.g., Tromp-Van Meerveld and Mcdonnell, 2006). Nevertheless, climatic features such as precipitation, solar radiation and temperature, acting at far larger spatial scale, also have an important influence (Grundmann, 2004; Griffiths et al., 2011; Convey et al., 2014). In addition to physical and chemical environmental influences, community assembly is also controlled by biological features such as dispersal, interaction (e.g., competition, predation), motility and reproduction (Ettema and Wardle, 2002; Webb et al., 2002).

Among exceptional ecosystems of the planet, Antarctic terrestrial environments are characterized by high winds, intense UV radiation, desiccation, and low temperatures. These physical stressors challenge Antarctic life (Kennedy, 1993; Convey, 1996; Wall and Virginia, 1999; Hogg et al., 2006; Cary et al., 2010) and, combined with physical isolation and geographical barriers (e.g., circumpolar oceanic and atmospheric currents), limit inter- and intra-continental connectivity and underlie the level of endemicity present in Antarctica today (Franzmann, 1996; Clarke et al., 2005; Adams et al., 2006; Barnes et al., 2006; Taton et al., 2006; Convey et al., 2008, 2009, 2014; Vyverman et al., 2010). Given the many differences in physical setting and adaptive requirements, as well as the scales of biological organization involved (e.g., Figure 2 in Peck, 2011), researchers have sought to understand the links between spatial diversity and functioning of Antarctic communities and the differences in comparison to other ecosystems (see Convey et al., 2014 for discussion). Detailed and spatially explicit knowledge of Antarctic biodiversity is essential to enable construction of a comprehensive framework for conservation planning (Hughes and Convey, 2010, 2012; Terauds et al., 2012; Convey et al., 2014; Chown et al., 2015), and to provide baseline data for ecological modeling and prediction (Gutt et al., 2012); however, our knowledge of microbial systems and functions is, at best, fragmented, both globally and in the Antarctic specifically (Tindall, 2004; Cary et al., 2010; Chong et al., 2013).

In this review, we collate current knowledge of Antarctic microbial diversity and biogeography. Adopting a similar approach to that of Martiny et al. (2006), we focus our discussion primarily on Antarctic prokaryotic spatial patterning, making reference to patterns inferred in Antarctic eukaryotic studies where appropriate. We do not assume that the prokaryotes exhibit the same ecological patterns as the eukaryotes, however, the latter have been relatively well-studied and provide a useful comparison. We identify gaps in current knowledge, along with limitations in the methodologies available. Our synthesis leads to the proposition of a new conceptual model to explain the mechanisms underlying species-function relationships in Antarctica, and the experimental framework required to provide such mechanistic insight based on empirical data.

## Macroecological Patterns in Antarctica

Antarctica has traditionally and pragmatically been divided into three biogeographic zones, the sub-Antarctic, maritime Antarctic, and continental Antarctic (Convey, 2013). The sub-Antarctic includes a ring of oceanic islands located between c. 45° and 55°S, close to the Antarctic Polar Frontal Zone (Convey, 2007b; Selkirk, 2007). These experience relatively higher precipitation and milder and much less variable temperatures in comparison to the maritime and continental zones, and host the most complex Antarctic terrestrial ecosystems. The maritime Antarctic includes the Scotia Arc archipelagos of the South Sandwich, South Orkney and South Shetland Islands and the majority of the Antarctic Peninsula southward to Alexander Island. Crytogamic fellfield is the most typical vegetated habitat along the coastline and associated low lying islands. In addition, vegetation “hotspots” can be found on the nitrogen-rich ornithogenic gelisols formed near seabird colonies or seal haul-out areas (Michel et al., 2006; Bokhorst et al., 2007). Finally, continental Antarctica comprises the eastern and southern parts of the Antarctic Peninsula, and the remainder of Antarctic continent. Terrestrial ecosystems within this region are restricted to small isolated “islands” of ice-free ground located mainly either in the low-lying coastal zones, or in the form of isolated nunataks and the higher altitudes of inland mountain ranges, with the striking exception of the McMurdo Dry Valleys in Victoria Land which cover an area of approximately 40,000 km^2^.

In recent years, large-scale spatial comparisons have refined our understanding and revealed a far greater complexity in the patterns of biogeography present in the terrestrial ecosystems of Antarctica than previously appreciated (Chown and Convey, 2007; Convey et al., 2008; Terauds et al., 2012). For instance, studies across a range of terrestrial macro- and micro- eukaryotic organisms (plants, algae, insects, springtails, mites, nematodes, tardigrades, rotifers) have revealed a strong division between the Antarctic Peninsula and the remainder of the continent (e.g., Maslen and Convey, 2006; Peat et al., 2007; Pugh and Convey, 2008; De Wever et al., 2009; Iakovenko et al., in press). Chown and Convey (2007) proposed that this distinction represented an ancient boundary analogous to the Wallace Line of south-east Asia, reflecting Antarctic historical contingency (the “Gressitt Line”). Separately, a strong localized diversity was also detected when comparing the genetic lineages of Antarctic microbial eukaryotic organisms across different locations (Lawley et al., 2004; Namsaraev et al., 2010). More recently, a spatial analysis of 38,854 entries and 1823 eukaryote taxa recorded in the Antarctic Biodiveristy Database (ABD)^1^ revealed 15 distinct ‘Antarctic Conservation Biogeographic Regions’ across Antarctic terrestrial environments (five within the classical maritime Antarctic region and 10 from the continental Antarctic; Terauds et al., 2012).

## Spatial Patterns of Prokaryotic Diversity

The elucidation of spatial patterns of organization in Antarctic eukaryotes provides an excellent opportunity for microbiologists to evaluate the degree to which prokaryotic biogeography in the Antarctic mirrors or differs from that of the eukaryotes, and to shed new light onto the functioning of Antarctic terrestrial ecosystems. If biogeographic processes in both major groups operate at similar spatial scales, then a homogenous set of mechanisms can be hypothesized to govern these processes, and a consistent response to environmental changes can be predicted. In contrast, the finding of distinct spatial patterns would be indicative of fundamental differences in, for instance, life history, survival strategies, or dispersal limitation. The latter would, further, have important implications for the planning of biosecurity and biodiversity management in Antarctica, including in the application of guidelines and protocols developed under the Environmental Protocol to the Antarctic Treaty and the definition of Antarctic Specially Protected Areas (ASPAs), as current practice has almost completely been built upon knowledge of macro-organisms such as vertebrates, invertebrates, and plants (Hughes et al., 2015).

Over the last decade, encouraged by improved technical and methodological capabilities, knowledge of the spatial scaling and the functional capabilities of Antarctic prokaryotic communities has started to increase. It is thus timely to review our knowledge of bacterial biogeography in Antarctica and to ask how spatial patterns influence ecological functions in the microbial communities of Antarctica.

## Site-specific Bacterial Diversity

### Airborne Diversity

Antarctica is an extremely windy place. Long distance inter-continental air mass movement has been shown to be a viable route for non-native propagules from Australia, South America, and South Africa to reach and potentially establish in Antarctica (Linskens et al., 1993; Marshall and Convey, 1997; Greenslade et al., 1999; Convey, 2005; Pearce et al., 2009). Locally, the magnitude and direction of air movement vary widely across Antarctica. However, strong and complex networks of aeolian exchange and interaction are apparent. For instance, the low-lying coastal regions of the Antarctic continent and Antarctic Peninsula periodically experience high velocity katabatic winds which may bring mineral dust from the continental interior (Turner et al., 2009; Pearce et al., 2010). It is not clear if this enables the transfer of viable propagules from the polar plateau to the coastal region, however, similar air movements have been documented in back trajectory analyses of air parcels studied microbiologically (Marshall, 1996; Hughes et al., 2004; Pearce et al., 2010; Bottos et al., 2014b). Additionally, the circumpolar coastal winds (circulating west to east) increase the mixing of air masses between the interior and coastal areas, and further facilitate inter-regional aeolian movement between different ice-free regions in Antarctica (Wynn-Williams, 1991; Reijmer et al., 2002; Parish and Bromwich, 2007).

The very limited aerobiological survey data currently available from the Antarctic Peninsula and continental Antarctic generally suggested low airborne bacterial diversity and a minimal contribution of local propagules into the aerosol (Hughes et al., 2004; Pearce et al., 2010; Bottos et al., 2014b). For instance, marine-related sequences constituted <10% of the airborne bacterial diversity detected at Halley V Research station on the Brunt Ice Shelf at the base of the Weddell Sea and at Rothera Point, to the west of the Antarctic Peninsula, despite substantial sea-spray influence in both locations. Separately, Bottos et al. (2014b) observed little overlap between the aerosol and soil bacterial diversity in the McMurdo Dry Valleys.

Overall, there was little similarity in bacterial diversity in the studies reported by Hughes et al. (2004), Pearce et al. (2010), and Bottos et al. (2014b). Although this might relate to differences in methodologies employed in each study, the differences might also be underlain by the environmental stresses faced in long duration airborne dispersal (e.g., Hughes et al., 2004; see also review by Pearce et al., 2009). High community variation was also detected when comparing the microbiota of aerosols collected in close proximity (e.g., ~2 km apart, Bottos et al., 2014b), further supporting strong spatial variation in Antarctic aerosols. However, a number of cyst forming and desiccation resistant genera such as *Frankia*, *Rubrobacter*, *Sphingomonas*, and *Paenibacillus* were found. These genera might form the core of an airborne bacterial community that is universal across Antarctica (Pearce et al., 2010; Bottos et al., 2014b).

### Soil Microbial Diversity

Recent Antarctic terrestrial microbiological studies using molecular approaches generally support the occurrence of highly specific community membership across space. For instance, in bacterial culture collections developed from nine distinct sites in the Antarctic Peninsula, and the Ronne, Maud, and Enderby sectors of continental Antarctica (Peeters et al., 2012), only 3.4% of the total isolates were common to more than one site. More generally, it has been estimated that <1% of total bacterial diversity is culturable in temperate environments (Hugenholtz, 2002), so these common isolates may represent an even smaller percentage of the overall diversity. In a similar report of highly localized bacterial distribution patterns derived using a culture-independent technique, Lee et al. (2012) reported that, in four cold desert habitats located within an 80 km radius in the McMurdo Dry Valleys, the proportion of rare phylotypes specific to only one site ranged between 48 and 72%.

At higher phylogenetic levels, such as phylum or class, the dominant membership of Antarctic soil bacterial communities is relatively consistent (e.g., Yergeau et al., 2007b; Pointing et al., 2009; Chong et al., 2012b), including common groups found in soil ecosystems globally such as Acidobacteria, Proteobacteria, Firmicutes, and Bacteroidetes (Janssen, 2006; Youssef and Elshahed, 2008). Nevertheless, in comparisons across different Antarctic regions, strong compositional differences become apparent. For example, soil from Antarctic Peninsula sites was dominated by taxa affiliated with Alpha-proteobacteria and Actinobacteria and had low representation of Bacteriodetes, while the reverse pattern was apparent in soil from the Ellsworth Mountains (Yergeau et al., 2007b). Separately, Actinobacteria contributed the largest proportion of the overall soil bacterial community in Victoria Land, more than double that detected in the former two locations (Bottos et al., 2014a, and references therein). Again, methodological differences may contribute to such observations, although it is notable that diversity variations are also apparent in comparisons of regional samples using standardized methodology (Yergeau et al., 2007b; Sokol et al., 2013).

Even greater variation was apparent in the ‘rare’ members of the community – those which make up less than 0.05% of the community composition. For instance, members of *Verrucomicrobia* and *Spirochaetes* were detected rarely in rhizosphere soil in the Antarctic Peninsula but were completely absent from mineral soils in the Antarctic Dry Valleys (Teixeira et al., 2010; Lee et al., 2012). Both these studies employed massively parallel next generation sequencing (NGS) techniques targeting similar 16S regions (V4–V5 vs. V3–V5) and reported high average sequence coverage at 90%. Assuming that the disparity in the community assembly between locations is not due to methodological variation, it might be a reflection of the different requirements and life history strategies of various microbial lineages.

## Environmental Selection vs. Geographical Isolation

Syntheses of studies of physiological adaptation and life history strategies of Antarctic organisms have suggested that the distribution of Antarctic terrestrial life is generally driven by abiotic environmental gradients in variables such as the availability of water or specific nutrients (Kennedy, 1993; Convey, 1996; Barrett et al., 2006a; Hogg et al., 2006; Convey et al., 2014). For example, the water gradient at Mars Oasis (Alexander Island, Antarctic Peninsula) leads to a clear separation between populations of *Mortierella* and *Serendipita*-like *Sebacinales*, *Tetracladium*, *Helotialian* fungi and black yeasts (Bridge and Newsham, 2009). Similar trends have also been observed in studies of soil arthropods, for instance with some mite species such as *Gamasellus racovitzai* and *Alaskozetes antarcticus* showing a stronger resistance to desiccation stress than others such as *Stereotydeus villosus*, while the length of the active season appears to be more strongly influenced by the moisture available in the environment for some species than others (Convey et al., 2003). Green algae including *Nostoc* spp. and *Gloeocapsa* spp. are sensitive to salinity and hence are usually absent from areas subjected to frequent windblown sea-spray (Broady, 1996). In addition, heavy metals including copper are detrimental to the growth and the cell wall structure of cyanobacteria and might thereby inhibit the distribution of the photosynthetic microbes in the Dry Valleys (Wood et al., 2008).

Although most such syntheses have been based on studies of Antarctic invertebrates and plants, similar findings are apparent in recent molecular studies of Antarctic soil bacterial communities (**Table 1**). For instance, in the Ross Sea region of continental Antarctica, Aislabie et al. (2008) found strong positive correlation between bacterial community diversity and soil pH and nutrient content. In the Dry Valleys of the same region, Lee et al. (2012) proposed that salt and copper content in the soil, along with altitude, were the major drivers of microbial community composition. Over a spatial gradient of a few kilometers in a coastal area of maritime Antarctica, Chong et al. (2012a) similarly reported that community structure was largely determined by pH and altitude. Magalhães et al. (2012) working near Darwin Mountain (Transantarctic Mountains) found different ion concentrations were the main driver of diversity. It is striking that none of these studies established strong distance decay or occupancy-distance relationships in bacterial community composition, consistent with the findings of a recent large-scale spatial study within the Transantarctic Mountains (Sokol et al., 2013). Based on spatially stratified sampling that spanned seven degrees of latitude, Sokol et al. (2013) showed that local edaphic gradients (e.g., pH and moisture) exerted stronger control over the bacterial community composition than was explained by spatial scaling alone. In comparison, however, spatial partitioning was prominent in the cyanobacterial community, potentially indicating differences in dispersal controls between cyanobacteria and the soil bacterial community.

**Table 1:** Major environmental parameters influencing terrestrial bacterial community composition.

Major environmental parameters^a^	Correlate with^b^	Microbiological approach	Region	Spatial range	Reference
pH	BCS	DGGE	Signy Island	<10 km	Chong et al., 2010
pH	BCS	NGS	Windmill Island	<100 km	Siciliano et al., 2014
pH	BCS	NGS	McMurdo Dry Valleys	<100 km	Van Horn et al., 2013
pH	BR and CS	TRFLP	Antarctic Peninsula	<10 km	Chong et al., 2012b
pH and EC	BCS	TRFLP	Scott Base	<1 km	O’Neill et al., 2013
pH and EC	BCS	Cloning	Ross Sea region	<100 km	Aislabie et al., 2008
pH and EC	BCS	TRFLP	McMurdo Dry Valleys	<100 km	Geyer et al., 2013
pH and copper	BCS	DGGE, TRFLP and Cloning	Alexander Island	<10 km	Chong et al., 2012a
pH and moisture	BCS	ARISA	Victoria Land	>100 km	Smith et al., 2010
pH, nitrate, temperature	BCS	DGGE	Cross regional study	>100 km	Yergeau et al., 2007c
Altitude and EC	BCS	ARISA	McMurdo Dry Valleys	<100 km	Lee et al., 2012
Carbon content	BR	TRFLP	McMurdo Dry Valleys	<100 km	Geyer et al., 2013
Carbon, nitrogen, and EC	BR	ARISA	Darwin Mountain	<5 km	Magalhães et al., 2012
Carbon, nitrogen, and moisture	BCS	DGGE	South Shetland Archipelago	<5 km	Ganzert et al., 2011
Carbon, nitrogen, and moisture	Microbial abundance	CFU counts	Cross regional study	>100 km	Yergeau et al., 2007c
Carbon, nitrogen, and chloride	BR	NGS	Windmill Island, Eastern Antarctica	<100 km	Siciliano et al., 2014

*^a^EC, electrical conductivity; ^b^BCS, bacterial community structure; BR, bacterial richness.*

A large-scale compilation of bacterial 16S rRNA gene sequence data retrieved from Antarctic soil habitats ranging from 45 to 78°S revealed that majority of the Antarctic soil habitats included were phylogenetically clustered (genetically closely related, see Webb et al., 2002), implying strong habitat filtering in the Antarctic terrestrial environment (Chong et al., 2012b). Souza et al. (2008) hypothesized that bacterial community homogenization in nutrient-depleted environments might be obstructed by low cell density, which could reduce the likelihood of horizontal gene transfer across the community. Additionally, environmental stress might further exert sympatric selective pressure in different micro-niches in the soil, promoting the prevalence of specialists in each ecotype. Such factors might underlie the detection of the highly specialized communities reported in various studies (Lee et al., 2012; Peeters et al., 2012). In a separate large-scale latitudinal survey in the Antarctic Peninsula/Scotia Arc region, Yergeau et al. (2007b) showed a significant latitudinal influence on the bacterial community composition of bare ground sites. However, for locations with moss/lichen cover, the effect of local vegetation cover far outweighed any influence of geographical isolation.

If a combination of soil edaphic parameters and nutrient availability is the main driving force for prokaryotic community assembly in harsh Antarctic environments, it is perhaps justifiable to postulate that taxonomic diversity in Antarctica should be lower in comparison to those of temperate and tropical regions. Additionally, the Antarctic bacterial community might resemble those of other cold desert habitats such as parts of the Arctic and high altitude montane regions. Detailed molecular microbial assessments of Antarctic terrestrial ecosystems have, in contrast, demonstrated that Antarctic soil environments, including those from true frigid desert soils, harbor broad lineages with flexible functions that are comparable to other ecosystems globally (Cowan et al., 2002, 2014; Cary et al., 2010). In comparison, strong regional variation in Cyanobacteria and Archaea distribution was observed when comparing the distributions of these taxa across different desert habitats (Bahl et al., 2011; Bates et al., 2011). Separately, examination of the global distribution of cold-adapted genera including *Polaromonas, Psychrobacter*, and *Exiguobacterium* suggested that the Antarctic species formed distinct mono- and/or paraphyletic clusters specific to Antarctica when compared with close representatives from other regions (Rodrigues et al., 2009; Darcy et al., 2011).

**FIGURE 1:**
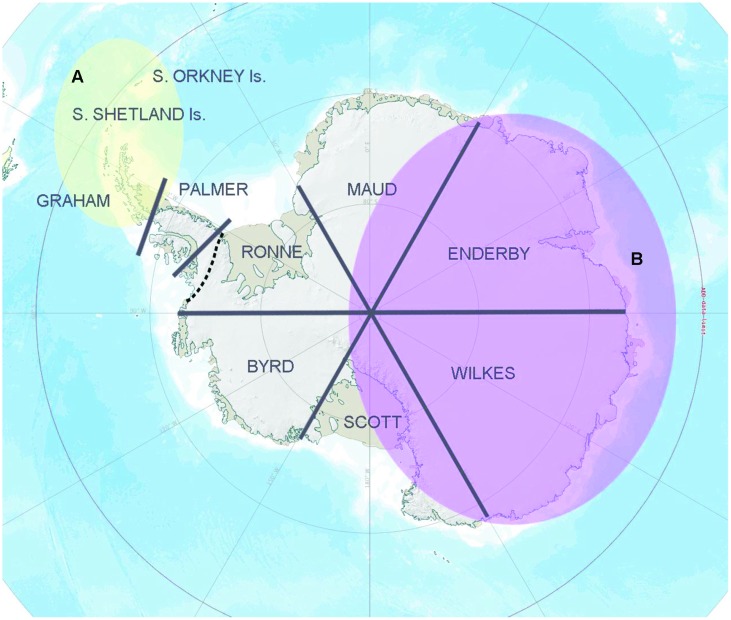
**Regional bacterial biogeography pattern based on the 16S rRNA gene data information.** A strong genetic separation was detected in the overall soil bacterial community composition and Bacteroidetes assemblages retrieved between zone A (yellow) and zone B (purple; Chong et al., 2012b), representing different sides of the “Gressitt Line” (dotted line). A similar pattern was observed in bacterial isolates affiliated with the genera *Flavobacterium* and *Arthrobacter* (Chong et al., 2013).

At a regional scale, geographical isolation clearly contributes to Antarctic microbial community diversification (Papke and Ward, 2004; Bahl et al., 2011). Indeed, simply by using the pragmatic and non-scientifically established geographical sectors of Antarctica outlined by Pugh and Convey (2008), Chong et al. (2012b) showed significant genetic separation in 16S rRNA gene sequences between soil bacterial communities obtained from the different sectors, a separation that was particularly apparent in *Flavobacterium* and *Arthrobacter* (**Figure 1**) although, again, such conclusions may be influenced by the application of inconsistent methodologies. However, the pattern found was also consistent with the Gressitt Line of Chown and Convey (2007), potentially suggesting the presence of a “universal” spatial constraint for both Antarctic higher and lower organisms.

Overall, we suggest that the spatial organization of Antarctic prokaryotic communities is highly dependent on the spatial scale studied. At small to moderate spatial scales (100 m–1000 km), community assembly is highly sensitive to the heterogeneity in local physicochemical parameters. At regional scale (>1000 km), however, the disparity in membership may reflect stronger influence of historical contingency (sympatric speciation) and dispersal limitations than geomorphological variation *per se.*

## Issues and Limitations of Antarctic Prokaryotic Biogeography

Various limitations currently hamper the interpretation of spatial patterns in Antarctic prokaryotic communities. We highlight some of the major hurdles faced here.

### Species Conundrum

Clear definition of species or taxonomic unit is a major prerequisite of efforts to characterize spatial patterns of distribution. As prokaryotic microorganisms, along with many algae and fungi, are generally cryptic (morphologically indistinguishable) and metabolically flexible, the distinction between different “species” is commonly based on variation in a phylogenetic marker (e.g., the 16S rRNA gene). The use of the phylogenetic markers has several advantages (e.g., they are evolutionarily conserved in all prokaryotes, lateral transfers of the genes are rarely reported, large databases are available, and the need for pure isolates is removed; Hugenholtz, 2002; Cole et al., 2009; Fierer and Lennon, 2011), although the inference of ecological role using phylogenetic markers alone is not always straightforward. For instance, variability between bacterial genotype and phenotype is well-documented (Fuhrman, 2009; Priest et al., 2012). Indeed, the level of variability in phylogenetic markers is itself variable across taxa (De Wever et al., 2009; Fraser et al., 2009), raising the often ignored problem that there is no clear or universally accepted level of variation required for the definition of a distinct species (Green and Bohannan, 2006), either within a particular lineage or across groups more generally. In Antarctic bacterial studies to date, a range of 97–99% cut-off points in sequence homology in the 16S rRNA gene has been applied (Aislabie et al., 2008; Newsham et al., 2010; Pearce et al., 2010; Peeters et al., 2011a). One alternative approach to overcome this problem is to define the phylogenetic relationship using the “metagenomics binning” strategy of Sharon et al. (2013). However, the assembly of short metagenomic fragments can itself be erroneous as it is sensitive to the occurrence of dispersed repeats. This is further exacerbated by the presence of closely related but heterogeneous genomes common in natural microbial populations. Nevertheless, these issues are being addressed through improvement in sequencing platforms and chemistry (e.g., Illumina TruSeq, Pacific Biosciences sequencing) that permit the generation of long and structurally explicit reads (Quail et al., 2012; Sharon et al., 2015).

### Technical Limitations

Over the past century, considerable progress has been made in the understanding of prokaryotic diversity in Antarctica. In the early 1900s the isolation of microorganisms quickly disproved the general perception that Antarctica is “sterile and devoid of life,” and it was already observed that the isolates were phenotypically similar to those from tropical and temperate regions. In the 1990s, by comparing Antarctic isolates with their closest relatives from elsewhere, a few studies started to suggest that the former were genetically distinct (Franzmann and Dobson, 1993; Franzmann, 1996). However, the true spectrum of prokaryotic life in Antarctica still lay beyond the reach of scientific study owing to the lack of isolates and ability to develop cultures.

This started to change when molecular microbiological profiling and cloning techniques came into play (Nocker et al., 2007). Antarctic soil profiling is now typically revealing a high diversity of microbial life, including in less studied habitats such as hypolithic and endolithic environments (Pointing et al., 2009; Cowan et al., 2010). Relatively recently, the advent of massively parallel NGS is further improving our knowledge of the functionality and diversity of Antarctic prokaryotic communities (Bates et al., 2011; Pearce et al., 2012; Tytgat et al., 2014; Kim et al., 2015). It is important to highlight that interpretation of NGS data is highly dependent on the quality of sequence assembly, OTU assignment and annotation. As suggested earlier, the key issue is to produce high quality long reads for downstream bioinformatics analysis.

The wealth of new data has improved the interpretation of ecological dynamics and diversity in Antarctic ecosystems (Cary et al., 2010; Cowan et al., 2014). It is, however, important to realize that diversity patterns have commonly been inferred by comparing preceding reports from similar habitats, or the collation of a series of local data for regional interpretation. Such approaches have usually involved studies with inconsistent methodologies and hence need to be handled with care.

Our understanding of the distribution of the rare members (contributing <0.05% overall diversity – for the purposes of this paper we consider 0.05% as ‘rare,’ although there appears to be no accepted definition in microbiological studies) of the Antarctic biosphere remains particularly weak. Although high-throughput NGS approaches provide a better option for capturing these rare community members than clone library and profiling methods, their short-read length is only suitable for informing on the presence of rare species and provides little information about their ecological role and functions (Sharon et al., 2015; Youssef et al., 2015).

Further, it is known that DNA/RNA extraction techniques may be selective toward purifying the genetic signature of taxa with weak cell walls (Hirsch et al., 2010). It is also unclear how representative the extracted DNA/RNA is, as the mechanism of interaction between soil, DNA and RNA is poorly understood (Lombard et al., 2011). For example, legacy DNA and RNA may contribute a substantial fraction of the detected gene signature in Antarctic soil due to enhanced preservation under the cold and arid environmental conditions (Chong et al., 2013; Cowan et al., 2014). The requirement for application of PCR, especially in cloning, DNA profiling and targeted metagenomics approaches, also introduces potential bias into the downstream interpretation, as sequences with high affinity to the primer sequences may be preferentially amplified in this process (Taberlet et al., 2012).

It is intuitively obvious that the application of one approach will not be sufficient to provide a complete picture of the prokaryotic community in Antarctica (or elsewhere). As the available technology advances, detailed systems biology approaches linking the diversity, RNA transcript (metatranscriptomics), metabolite (metabolomics), and protein (metaproteomics) signatures will be required to examine the contribution of richness and diversity to the ecological services provided by Antarctic prokaryote communities (Zengler and Palsson, 2012).

### Lack of Spatial Coverage

Microbiological studies in Antarctica have taken place since the earliest expeditions exploring the continent (Ekelöf, 1908). Until recent decades, studies have been culture-based and focused on describing the novelty of isolated strains, and to relating apparent diversity to local environmental features (e.g., Holdgate, 1977; Franzmann and Dobson, 1993). Historically such studies, which generally do not require elaborate systematic spatial sampling methodologies, have often been opportunistic in nature, depending on the presence of particular researchers with appropriate specialist skills at any given location and season (Chown and Convey, 2007). Consequently, historical microbiological work has been heavily spatially biased to areas accessible from particular research stations and, in particular, to a few relatively well-sampled regions in the Scotia Arc, west Antarctic Peninsula, McMurdo Dry Valleys and the coastal region of Wilkes Land (Smith et al., 2006; Aislabie et al., 2008; Chong et al., 2012b, 2013; Dennis et al., 2013).

The global ubiquity theory postulates that the dispersal potential of microbes (including prokaryotes) is less confined by geographical barriers than is the case for larger organisms (Baas Becking, 1934; Finlay, 2002). While the universal applicability of this theory is increasingly questioned (Martiny et al., 2006; Woodcock et al., 2007), studies such as De Wever et al. (2009) and Bahl et al. (2011) do appear to suggest strongly that the Antarctic microbiota is more distinct than that of the other continents globally, supporting the effectiveness of the barriers isolating the Antarctic continent.

There is a general consensus that the influence of abiotic factors in population selection is expected to be amplified under harsh Antarctic conditions (Barrett et al., 2006a; Hogg et al., 2006). Perhaps as a result, most microbial biogeographical studies to date in Antarctica have given strong emphasis to the role of local environmental drivers in defining community composition and structure (Barrett et al., 2006b; Chong et al., 2010; Ganzert et al., 2011; Magalhães et al., 2012), and few have considered the spatial patterns, controls and functions that might be apparent at a larger sampling scale in Antarctica.

While lack of spatial coverage is a limitation that has been identified as being common to all other major Antarctic taxonomic groups (Chown and Convey, 2007, 2012; Peat et al., 2007; Convey et al., 2012; Terauds et al., 2012), the limitation is more severe in the prokaryotes than in eukaryotic groups. Placed this in context, at present bacteria and archaea together contribute less than 6% of the total records available in the ABD^2^ (accessed 9 August, 2015). However, spatial issues are now gaining increasing attention, and have formed an integral part of recent scientific initiatives of several national operators such as the United Kingdom (Ecosystems Programme^3^), Australia (Terrestrial and Nearshore Ecosystem programme^4^) and New Zealand (New Zealand Terrestrial Antarctic Biocomplexity Survey^5^). The need for increasingly close cooperation in the form data of sharing, sampling coordination and field support has been identified clearly in the recent Scientific Committee on Antarctic Research ‘Antarctic and Southern Ocean Horizon Scan’ (Kennicutt et al., 2014a,b). With the ever-increasing data becoming available, more light will be shed on the effect of spatial scaling on Antarctic biotas.

## Ecological Functions and Biogeography of Antarctic Bacterial Communities

There is general agreement on there being a positive correlation between species diversity and functional richness: the greater the number of species, the greater the functional richness of a community, or alternatively, fewer species being present leads to a lack of functional redundancy (Peterson et al., 1998). In a highly diverse ecosystem, the likelihood of overlapping ecological function between species increases, creating communities that may be functionally similar despite involving different combinations and proportions of individual species.

Due largely to the absence of the major soil eukaryotic groups and the lack of biotic interactions (Hogg et al., 2006), functional redundancy is often assumed and predicted to be low in Antarctic soil (Convey, 2007a). If so, then each species in a given Antarctic community might be responsible for the provision of a distinct and irreplaceable ecological function. This idea is in congruent with the observation of low nematode species count and low cross-biome functional diversity in Antarctic Dry Valley soils (Wall and Virginia, 1999; Fierer et al., 2012). As ecological resilience is built upon the functional diversity of the ecosystem, habitats hosting extremely low biodiversity, as has been suggested for some inland dry valley ecosystems in Antarctica (Wall and Virginia, 1999; Hodgson et al., 2010; Fernandez-Carazo et al., 2011; Peeters et al., 2011b), might be particularly vulnerable to environmental disturbance (Tiao et al., 2012). Combining the concepts of low biodiversity and limited function, the detection of regional bacterial biogeography within Antarctica may also imply the presence of regional-specific variation in functional capability in the continent’s soils.

Yergeau et al. (2007a, 2009) provided evidence of a close relationship between phylogenetic diversity and functional gene distribution in Antarctic soil. Using a combination of Geochip microarray and real-time PCR approaches, they suggested that a significant proportion of the variation in functional diversity observed along a latitudinal transect in fellfield soils between the Falkland Islands (51°S), Signy Island (60°S), and Anchorage Island (67°S) could be explained by geographical location, with the three locations harboring phylogenetically distinct soil bacterial communities (Yergeau et al., 2007b).

Chan et al. (2013) assessed the functional diversity of the McKelvey Valley in the McMurdo Dry Valleys, using a much updated Geochip microarray. They established, in contrast to the previous study, that Antarctic hypoliths, chasmoendoliths and bare soil hosted significantly different functional diversity, with the former including a greater range of stress-response related genes, and the latter including specific genes affiliated with hydrocarbon transformation and lignin-like degradation pathways. However, little functional variation was detected between the five bare soil samples examined, despite the samples having previously been shown to support heterogeneous phylogenetic diversity (Pointing et al., 2009). Similarly, Yergeau et al. (2012) showed that the majority of members of the Antarctic Peninsula soil community were functionally similar (functional generalists) despite apparent differences in microbial diversity particularly between vegetated and non-vegetated sites (Yergeau et al., 2007b), potentially indicating some level of redundancy in the Antarctic soil system. The number of functional genes detected in these soils was also surprisingly high in absolute terms, with some sites in the McMurdo Dry Valleys harboring functional richness comparable to temperate and tropical forests (Fierer et al., 2012).

Several recent studies applying newly available molecular approaches have drawn conclusions relating to microbial diversity that are contrary to the common belief that reduced biodiversity in Antarctica equates to a functionally challenged ecosystem (Cowan et al., 2002; Pearce et al., 2012; Stomeo et al., 2012). This highlights the need to develop studies examining microbial interactions, such as communication (e.g., quorum sensing and quenching) and competition in these systems. For instance, *Clostridium* and *Flavobacterium*, which usually dominate nutrient-rich habitats such as penguin rookeries (Aislabie et al., 2009), penguin guano (Zdanowski et al., 2005) and the rhizosphere (Teixeira et al., 2010) were also part of the core phyla detected in extremely arid mineral soils (Tiao et al., 2012). These lineages may play a pivotal role in nutrient release in the event of chance deposition of nutrients (e.g., in the form bird perches or seal carcasses) in the Dry Valleys (Cary et al., 2010; Tiao et al., 2012). In parallel, Hughes and Lawley (2003) detected the fungal genus *Verticillium*, rarely found in saline habitats, in gypsum encrusting rocks on Alexander Island in the maritime Antarctic. One explanation for the detection of such “unusual” taxa might be that the low competition in these less diverse environments facilitated greater success of “chance colonization” for rare species, allowing them to develop greater flexibility and occupy niches that would typically be occupied by other specialists in more diverse systems (Chase and Myers, 2011). In addition, Székely et al. (2013) suggested that species sorting is more prominent in competitive environments.

A meta-analysis of studies examining diversity–function relationships (Nielsen et al., 2011) concluded that species diversity and functional properties in soil systems did not have a simple linear relationship, rather often showing idiosyncratic patterns. They further concluded that species traits were more important in controlling functionality in the ecosystem than richness *per se*. This would suggest both that loss of an individual species may not always translate into a detrimental effect on ecological function, and that the absence of a species with an important trait will be catastrophic to the maintenance of the ecosystem. This is consistent with the argument of Konopka et al. (2014) that, while microbial community composition is in constant flux, functionality can remain steady as long as the function is maintained by populations within the community.

**FIGURE 2:**
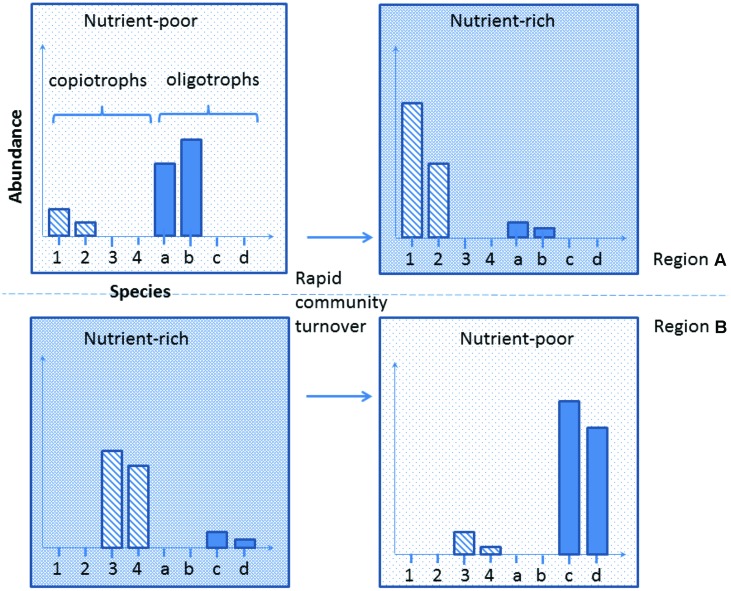
**A representation of Antarctic bacterial community dynamics in response to external environmental perturbation.** We believe that the Antarctic soil system harbors diverse functional traits that are preferentially selected based on suitability for the contemporary environmental conditions. Major environmental alteration may result in currently rare species being selected for and a major community compositional shift occurring. Note that habitats from different Antarctic regions may harbor different species with similar traits (upper vs. lower row).

**FIGURE 3:**
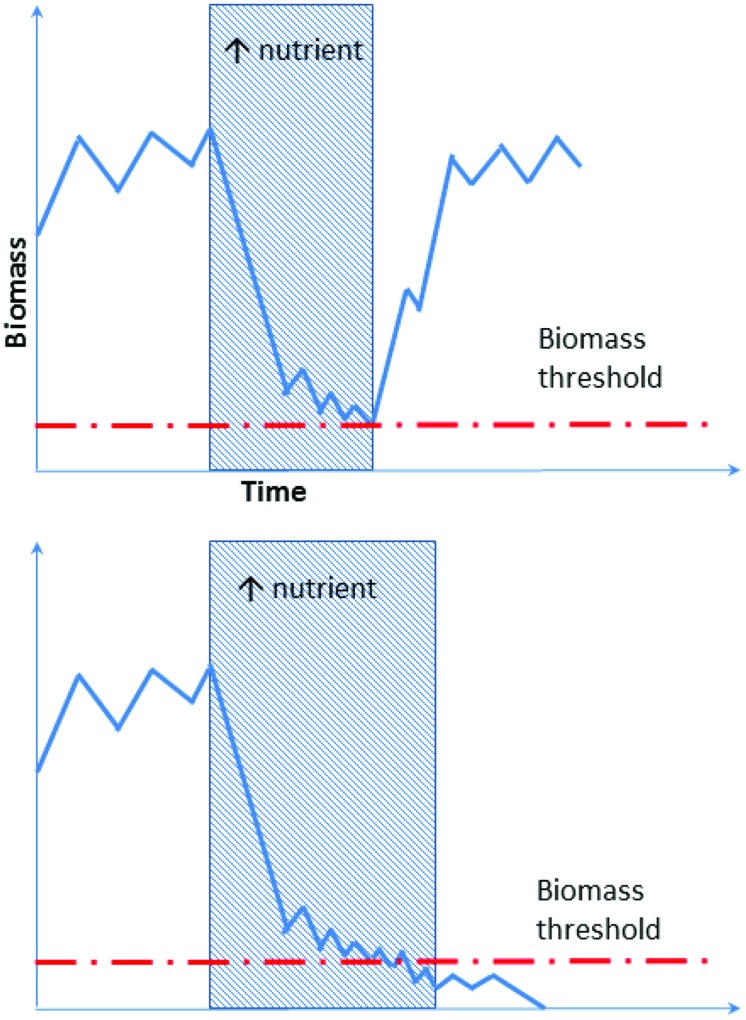
**Schematic illustration of an oligotroph’s response to alteration in local nutrient content.** The oligotroph is suppressed periodically when large amounts of nutrients are available. Biomass then returns to the original level when the nutrients become depleted by the copiotroph, promoted by environmental change. In the event of prolonged nutrient alteration oligotrophs may drop below the biomass threshold (lower graph), and it will not recover even if nutrient levels returns to the original state.

Developing this concept further, and integrating the increasing reports of bacterial regionalization within the Antarctic (Yergeau et al., 2007b; Chong et al., 2013; Sokol et al., 2013), we propose here a new conceptual model to explain the mechanism underlying species-function relationships in Antarctica. The Antarctic soil ecosystem is supported by a highly diverse but region-specific bacterial community. For instance, nutrient-rich (e.g., penguin rookeries) and nutrient-poor (e.g., barren soil) environments from different Antarctic regions contain both copiotrophs (high nutrient requirement, e.g., *Flavobacterium* spp.) and oligotrophs (low nutrient requirement, e.g., *Acidobacterium* spp.; Fierer et al., 2007; Aislabie et al., 2008; Chong et al., 2010; Bottos et al., 2014a). Soil samples obtained across different regions exhibit distinct community memberships with reference to these groups, but the phylogenetic similarity of their members is greater within the same biogeographic region than it is between regions (**Figure 2**, comparing upper and lower panels). In any particular system, the biomass of the copiotrophs and oligotrophs is dependent on the ecological characteristics of the habitat present. Nutrient-poor habitats host a greater percentage of oligotrophs such as Acidobacteria that convert recalcitrant carbon such as xylan (from autotrophs) and pectin (from wind-blown plant materials) into labile carbon (Bokhorst et al., 2007; Ward et al., 2009), while copiotrophs such as some Bacteroidetes dominate nutrient-rich sites, degrading the available high molecular weight organic carbon (Zdanowski et al., 2005; Aislabie et al., 2008; Chong et al., 2010). Changes in local environmental conditions, such as deposition of nutrients through aeolian transfer, or loss through leaching, can trigger rapid community turnover to match the new functional requirement (Saul et al., 2005; Barrett et al., 2006a; Tiao et al., 2012; Dennis et al., 2013; **Figure 2**). If such community compositional shifts involve specialists (rare species with unique traits) being lost or reduced below a critical biomass level, this may become a limiting factor in responding to subsequent changes (**Figure 3**).

We acknowledge that this hypothesis could be difficult to test under normal field conditions due to the technical limitations applying to currently available molecular microbiology approaches, such as detection limits (for rare biosphere <0.05%) for both diversity and function and problems in discriminating the functions of individuals from various populations of the same community. Nevertheless, it was evident from a field study by Tiao et al. (2012) that rapid compositional shift in in response to nutrient enrichment by a seal carcass was detectable in the McMurdo Dry Valleys. One practicable approach to testing this would be to conduct detailed functional quantification in a series of microcosm experiments (cf. Newsham and Garstecki, 2007), analyzing the outcomes using long metagenomic reads (Sharon et al., 2015). Each microcosm would encompass different combinations of phylogenetically distinct microbial isolates with known function in order to represent a diversity gradient. Ecological thresholds could then be determined by comparing the minimum biomass of any given specialist required before a drop (‘step change’) in any ecological function is detected when growth conditions are altered.

## Conclusion

Over the last decade, rapid advances in molecular methodologies and progressive improvement in sampling strategies have started to realize some of the vast potential of Antarctic microbiology. Despite continuing restrictions in spatial coverage, Antarctic microbiologists are now increasingly confident that Antarctic soil ecosystems harbor a rich bacterial community performing versatile ecological functions (Cowan et al., 2002; Pearce et al., 2012; Chan et al., 2013). Based on recent molecular studies, it is clear that the functional capability of Antarctic soil communities is not simply linearly related to species richness, and considerable functional overlap has been observed between species (Yergeau et al., 2012; Chan et al., 2013). This is an important paradigm shift from the long-held view of simple ecosystems with low functional redundancy typifying Antarctica (Wall and Virginia, 1999).

Recent studies also demonstrate that the Antarctic soil microbial ecosystem is flexible and capable of rapid community adjustment in response to external environmental fluctuation (Tiao et al., 2012; Dennis et al., 2013). Such functional resilience may be a result of phenotypic plasticity of Antarctic biota and millions of years of adaptive selection. Nevertheless, we propose that community organizational shifts in response to perturbation are limited by the threshold biomass of the often rarer species that provide important functions required under contemporary environmental conditions (**Figure 3**). This, however, does not mean that the generalists forming the dominant biosphere are unresponsive to the environmental changes. For instance, rapid ecological drift was found to affect both prevalent and rare phyla in a multi-year mummified seal transplantation experiment conducted in the McMurdo Dry Valleys (Tiao et al., 2012).

Building on the observation of highly specific and localized patterns of bacterial biodiversity in community membership, and the presence of bacterial zonation or regionalization within Antarctica we suggest that, under comparable environmental conditions, the “limiting species” for ecological function will not be the same across different Antarctic regions.

Our model has important implications both to the direction of future research and to biosecurity management of Antarctic microbial ecosystems. First, it is important to understand how cross-trophic interactions are maintained under relevant spatial scales for both the prokaryotic and eukaryotic elements of the Antarctic terrestrial ecosystem. For instance, we now understand that, at a superficial scale, the Gressitt Line boundary may be applicable to both Antarctic macro- and microbiotas, but it is not clear whether parallel ecosystems across this boundary display similar or different trophic networks.

Second, acknowledging that each biogeographical region comprises phylogenetically distinct communities, it is imperative to identify the different key limiting species that determine functional resilience at different scales of spatial organization. However, given that functionally limiting species are often minority community elements, it can be challenging to detect their presence. As a further complication, the molecular signature of target species can potentially be masked by legacy DNA or RNA preserved under cold and arid Antarctic conditions. There is also currently a lack of knowledge of biomass or abundance thresholds required to sustain “specialist” populations. In order to generate greater understanding, there is a pressing need to extend the spatial coverage of microbial research across Antarctica, and the temporal sampling of field manipulation studies similar to those performed by Yergeau et al. (2012) and Dennis et al. (2013). Additionally, research should also focus on the evaluation of varying responses of communities in each distinct Antarctic biogeographic region to environmental variability and change, the introduction of non-native microbiota, and other anthropogenic impacts (Tin et al., 2009; Cowan et al., 2011; Chown et al., 2012). In conclusion, currently available evidence generally supports the proposition that Antarctic prokaryotes display large-scale regional biogeography similar to the patterns detected in eukaryotic groups. This allows a pragmatic comparison of the prokaryote and eukaryote spatial scaling and spatial patterns. Current functional assessments also point to the likelihood of functional redundancy existing in Antarctic prokaryotic communities. Nevertheless, it is clear that several key pieces of the puzzle are still missing, including the lack of spatially explicit information, and data on the genetics and functions of the rarer members of the Antarctic microbial communities. These gaps can be addressed in part through developing coordinated fundamental microbiology surveys across Antarctica, and complementary functional assessments through mesocosm studies.

## Conflict of Interest Statement

The authors declare that the research was conducted in the absence of any commercial or financial relationships that could be construed as a potential conflict of interest.

## Footnotes

1 http://data.aad.gov.au/aadc/biodiversity/

2 https://www1.data.antarctica.gov.au/aadc/biodiversity/taxon_drilldown.cfm

3 http://www.antarctica.ac.uk/bas_research/our_research/current/programmes/ecosystems/

4 http://www.antarctica.gov.au/science/terrestrial-and-nearshore-ecosystems-environmental-change-and-conservation

5 http://www.ictar.aq/nztabs.cfm
